# Many-body theory of positron binding to polyatomic molecules

**DOI:** 10.1038/s41586-022-04703-3

**Published:** 2022-06-22

**Authors:** Jaroslav Hofierka, Brian Cunningham, Charlie M. Rawlins, Charles H. Patterson, Dermot G. Green

**Affiliations:** 1 School of Mathematics and Physics, Queen’s University Belfast, Belfast, UK; 2 School of Physics, Trinity College Dublin, Dublin, Ireland

**Keywords:** Exotic atoms and molecules, Theoretical physics, Electronic structure of atoms and molecules

## Abstract

Positron binding to molecules is key to extremely enhanced positron annihilation and positron-based molecular spectroscopy^1^. Although positron binding energies have been measured for about 90 polyatomic molecules^1–6^, an accurate ab initio theoretical description of positron–molecule binding has remained elusive. Of the molecules studied experimentally, ab initio calculations exist for only six; these calculations agree with experiments on polar molecules to at best 25 per cent accuracy and fail to predict binding in nonpolar molecules. The theoretical challenge stems from the need to accurately describe the strong many-body correlations including polarization of the electron cloud, screening of the electron–positron Coulomb interaction and the unique process of virtual-positronium formation (in which a molecular electron temporarily tunnels to the positron)^1^. Here we develop a many-body theory of positron–molecule interactions that achieves excellent agreement with experiment (to within 1 per cent in cases) and predicts binding in formamide and nucleobases. Our framework quantitatively captures the role of many-body correlations and shows their crucial effect on enhancing binding in polar molecules, enabling binding in nonpolar molecules, and increasing annihilation rates by 2 to 3 orders of magnitude. Our many-body approach can be extended to positron scattering and annihilation γ-ray spectra in molecules and condensed matter, to provide the fundamental insight and predictive capability required to improve materials science diagnostics^7,8^, develop antimatter-based technologies (including positron traps, beams and positron emission tomography)^8–10^, and understand positrons in the Galaxy^11^.

## Abstract (web summary)

A many-body theory of binding interactions between positrons and polar and nonpolar molecules is developed, showing agreement with experimental data up to within 1%.

## Main

Pioneering technological developments have enabled the trapping, accumulation and delivery^8–10^ of positrons for study of their fundamental interactions with atoms and molecules^1,12^, and the formation, exploitation and interrogation of positronium (Ps)^13,14^ and antihydrogen^15,16^. The ability of positrons to annihilate with atomic electrons forming characteristic γ-rays makes them a unique probe over vast length scales, giving them important use in, for example, materials science for ultrasensitive diagnostics of industrially important materials^7,8^, medical imaging (positron emission tomography)^17^ and astrophysics^11^.

Proper interpretation of the materials science techniques and the development of next-generation antimatter-based technologies rely on an accurate understanding of the fundamental interactions of positrons with atoms and molecules. Substantial progress has been made developing ab initio theoretical understanding of positron–atom interactions^1,12,18–21^. Yet, for molecules, clusters and condensed matter, many basic aspects of positron–matter interactions remain poorly understood, and predictive capability is lacking. A notable example is the open fundamental problem of positron binding to molecules. Observation of energy-resolved annihilation spectra have enabled measurement of positron binding energies (ranging from a few to a few hundred meV) for more than 90 molecules^2–6^. The majority of these (approximately 60) are nonpolar or weakly polar species, such as alkanes, aromatics, partially halogenated hydrocarbons, alcohols, formates and acetates. By contrast, ab initio calculations have been performed predominantly for strongly polar molecules^1^ (though we note recent model calculations for polar and nonpolar molecules)^22,23^. Only six species have been studied both experimentally and with ab initio theory, via configuration interaction (CI)^24–28^ and ‘any particle molecular orbital’ (APMO)^29^ approaches: carbon disulfide CS_2_, acetaldehyde C_2_H_4_O, propanal C_2_H_5_CHO, acetone (CH_3_)_2_CO, acetonitrile CH_3_CN, and propionitrile C_2_H_5_CN^1^. For these, the sophisticated CI and APMO approaches proved deficient, greatly underestimating the experimental binding energies, agreeing to at best greater than approximately 25% (for acetonitrile, theory^28^: *ε*_b_ = 136 meV, versus experiment^5^: *ε*_b_ = 180 meV), and failing to predict binding in nonpolar CS_2_ (versus experiment: *ε*_b_ = 75 meV)^4^ (see below). Also, the considerably larger positron–molecule binding energies compared to electron–molecule ones (that is, negative ion states)^4,6^ are not quantitatively understood.

**Table 1:** Calculated positron–molecule binding energies

					Present many-body theory (meV)					Other calculations (meV)		
	*μ* (D)	*α* (Å^3^)	*I* (eV)	HF	*Σ*^(2)^	*Σ*^*GW*^	*Σ*^*GW*+*Γ*^	*Σ*^*GW*+*Γ*+*Λ*†^	Exp.^‡^	Benchmark	CI	APMO
**Polar molecules**												
LiH	5.9	3.50	8.3	130	434	518	1,291	1,106 1,038 **1,060**	–	1,043 (ECG)^40^	463^24^	–
Formaldehyde	2.3	2.43	11.2	0.3	9	10	51	27 25 **28**	–	25 ± 3 (QMC)^42^	15^26^	3^29^
Acetonitrile	3.9	4.24	12.6	15	120	109	301	210 195 **207**	180 ± 10	–	136^28^	65^29^
Propionitrile	4.1	5.90	12.4	16	140	129	341	245 230 **243**	245 ± 10	–	164^25^	–
Acetone	2.9	5.75	10.2	3	67	69	221	147 138 **152**	174 ± 10	–	96^28^	36^29^
Propanal	2.5	5.70	10.4	1	44	45	170	108 100 **108**	118 ± 10	–	58^26^	–
Acetaldehyde	2.7	4.12	10.6	2	35	38	135	86 81 **89**	88 ± 10	–	55^28^	16^29^
Formamide	3.7	3.68	11.0	12	105	109	255	186 178 **189**	~200*	–	–	–
**Nonpolar molecules**												
CS_2_	0	8.00	10.5	<0	<0	<0	171	68 46 **63**	75 ± 10	–	<0^27^	–
CSe_2_	0	10.7	9.7	<0	9	<0	276	139 101 **131**	–	–	18^27^	–
Benzene	0	9.85	9.5	<0	11	2	252	120 92 **116**	150	–	–	–

Dipole moment *μ* from ref. ^55^; isotropic polarizabilities *α* and ionization energies *I* calculated at the *GW* level (see Extended Data Table 1 for anisotropic polarizabilities). Binding energy calculations are presented in the Hartree–Fock (HF), *Σ*^(2)^ (bare-polarization) and *GW*@BSE (bare-polarization plus screening and electron–hole corrections) approximations, and additionally including virtual-Ps formation *Σ*^*GW*+*Γ*^ and the positron–hole ladder contribution *Σ*^*GW*+*Γ*+*Λ*^.

^†^For *Σ*^*GW*+*Γ*+*Λ*^, the first (second) number is that using bare (dressed) Coulomb interactions in the *Γ* and *Λ* blocks, and the third (our most sophisticated calculation, in bold) additionally uses *GW* energies in the diagrams. Their difference gives a measure of the theoretical uncertainty.

^‡^Experimental values from refs. ^1,4,5^, except for formamide.

*Experimental value for formamide is preliminary (J. R. Danielson, S. Ghosh & C. M. Surko, unpublished material).

Other calculations: ECG, explicitly correlated Gaussian; QMC, quantum Monte Carlo; CI, configuration interaction; APMO, any-particle-molecular orbital, at the best, ‘REN-PP3’ level. See also Fig. 2 for a graphical comparison.

For these molecules, vibrational and geometry relaxation effects are known to provide only a few per cent correction to fixed-nuclei calculations of binding energies and wavefunction densities^1,28–32^: for example, for acetaldehyde, acetone and acetonitrile (all considered in this work) the vibrational averaging correction was approximately 1–5%^28^. The theoretical difficulty lies in the need to identify and accurately describe strong many-body correlations that dominate the positron–molecule system. A powerful method that can fully account for the important correlations in a natural, intuitive and systematically improvable way is many-body theory^19,33–39^.

Here we develop the many-body theory of positron interactions with polyatomic molecules. We quantify and delineate the role of the correlations including polarization of the molecular electron cloud, screening of the positron–electron Coulomb interaction, and the unique process of virtual-Ps formation. We use the fixed-nuclei approximation and restrict to molecules with ionization energies larger than the Ps ground-state energy: for these, the Ps-formation channel is closed and the process is virtual, yet we will see that it has an important effect. After benchmarking its state-of-the-art computational implementation against explicitly correlated Gaussian (ECG) and quantum Monte Carlo (QMC) results for LiH and formaldehyde, we calculate binding energies and annihilation lifetimes for the six molecules for which both previous theory and measurements exist, finding excellent overall agreement. We additionally predict binding in formamide, CSe_2_, benzene and the primary nucleobases.

**Fig. 1:**
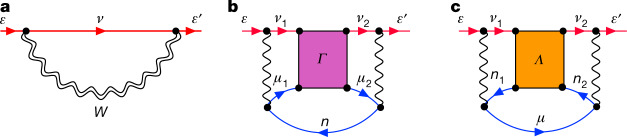
Main contributions to the positron–molecule self energy. **a**, The ‘*GW*’ contribution, which involves the positron Green’s function *G*_*ν*_ and the (dynamic part of the) screened Coulomb interaction *W*. It describes the positron-induced polarization of the molecular electron cloud and corrections to it due to screening of the electron–positron Coulomb interaction by molecular electrons, and electron–hole attractions. **b**, The virtual-Ps contribution *Σ*^*Γ*^, which includes the summed infinite ladder series (‘*Γ* block’) of screened electron–positron interactions. **c**, The positron–hole ladder series (the ‘*Λ* block’) contribution *Σ*^*Λ*^. Lines directed to the right (left) represent particles (holes) propagating on the *N*-electron ground-state molecule: red lines labelled *ε* represent the external positron state; other red (blue) lines represent positron (excited electron or hole) intermediate states that are summed over; single (double) wavy lines represent bare (screened) Coulomb interactions. See Methods and Extended Data Fig. 1 for details of their calculation via the BSE.

**Extended Data Fig. 1:**
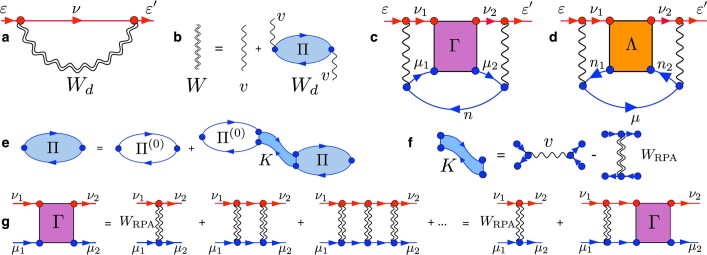
The main contributions to the positron–molecule self energy, including the two-particle propagators. **a**, The *GW* diagram involves the positron Green’s function *G*_*ν*_ and the dynamic part (due to the absence of an electron–positron exchange interaction) of the screened Coulomb interaction ***W***_d_ = ***vΠv*** (bold denotes operator form), where *Π* is the electron–hole polarization propagator (see **b**). It satisfies the BSE (**e**) with kernel *K* = *v* − *W*_RPA_ (**f**), where *W*_RPA_ = *v* + *W*_d,RPA_ is the screened electron–hole Coulomb interaction calculated in the random phase approximation (RPA). Setting *K* = 0 results in the bare polarization entering *W* only, and gives the *Σ*^(2)^ approximation, so-called as it is a second-order diagram in the electron–positron Coulomb interaction. Setting *K* = *v*, the direct part of the Coulomb interaction only, gives the random phase approximation (*GW*@RPA). Setting *K* = *v* − *v*_exch_—that is, including exchange which gives rise to interactions within the bubbles—yields the ‘time-dependent Hartree–Fock’ approximation (*GW*@TDHF). Using screened Coulomb interactions in the exchange term is the Bethe–Salpeter approximation (*GW*@BSE). **c**, The virtual-positronium contribution including the summed infinite ladder series of screened electron–positron interactions (‘*Γ* block’) shown in **g**. The contribution **d** contains the *Λ* block that is the ladder series of positron–hole interactions, which satisfies a linear integral equation of the same form as that shown in **g**.

The positron binding energy *ε* and bound-state wavefunction *ψ*_*ε*_ is found by solving the Dyson equation^33^ (*H*^(0)^ + *Σ*_*ε*_)*ψ*_*ε*_(**r**) = *εψ*_*ε*_(**r**), where *H*^(0)^ is the Hamiltonian of the positron in the Hartree–Fock field of the ground-state molecule, *Σ*_*E*_ is a nonlocal, energy-dependent correlation potential (irreducible self energy of the positron), and **r** is positron coordinate. It acts as an integral operator *Σ*_*E*_*ψ*(**r**) ≡ ∫(*Σ*_*E*_(**r**, **r**′))*ψ*(**r**′)d**r**′ and encapsulates the full complexity of the many-body problem. We calculate *Σ* via its expansion in residual electron–electron and electron–positron interactions, see Fig. 1. In Fig. 1a, the ‘*GW*’ self energy, *Σ*^*GW*^, describes the positron-induced polarization of the molecular electron cloud, and corrections to it owing to screening of the electron–positron Coulomb interaction by the molecular electrons, and electron–hole attractions (the Bethe–Salpeter equation approximation, *GW*@BSE). Figure 1b represents virtual-Ps formation^19,39^: it is denoted by *Σ*^*Γ*^ and involves the summed infinite ladder series of (screened) electron–positron interactions (the ‘*Γ* block’; see Extended Data Fig. 1). The infinite ladder series is important to the positron problem because successive diagrams in this series contribute to the positron–molecule self energy with the same sign, whereas for all-electron systems the series is sign alternating and gives a small overall contribution. We also consider the smaller positron–hole ladder series contribution, *Σ*^*Λ*^, shown in Fig. 1c. The construction of *Σ* and solution of the Dyson equation are detailed in Methods.

## Positron binding energies and lifetimes

**Fig. 2:**
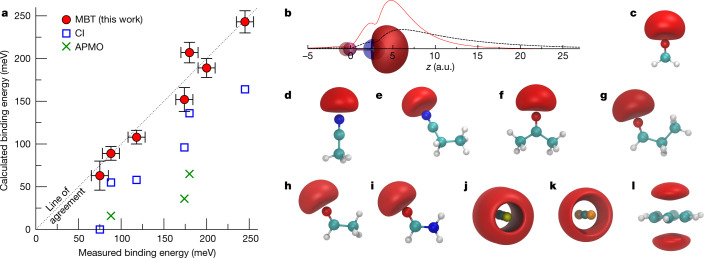
Positron–molecule binding energies and bound-state Dyson wavefunction densities. **a**, The comparison of the present many-body calculations (red circles) with experiment (error bars for the calculations show the largest difference between the three *Σ*^*GW*+*Γ*+*Λ*^ calculations in Table 1). Also shown are the CI and APMO calculations (blue squares and green crosses, respectively). MBT, many-body theory. **b**–**l**, Positron wavefunction densities. **b**, LiH, with Li atom at origin and H at approximately 3 a.u. along the molecular axis, showing the positron wavefunction density isosurface at 70% of the maximum (red lobe), the electron HOMO wavefunction density isosurface (blue lobe is the negative region at 40% of maximum, and brown is the positive region at 10% of the maximum). Also shown is the positron wavefunction calculated along the molecular axis in the Hartree–Fock approximation (black curve) and at the *Σ*^*GW*+*Γ*+*Λ*^ level of many-body theory (red curve). **c**–**i**, The positron wavefunction density isosurfaces at 80% of maximum for formaldehyde (**c**), acetonitrile (**d**), propionitrile (**e**), acetone (**f**), propanal (**g**), acetaldehyde (**h**), and formamide (**i**). **j**–**l**, Nonpolar molecules with isosurfaces at 90% of maximum CS_2_ (**j**), CSe_2_ (**k**), and benzene (**l**). a.u., atomic units. Source data

Table 1 shows our calculated binding energies at successively more sophisticated approximations to the correlation potential: Hartree–Fock, *Σ*^(2)^ (bare polarization), *Σ*^*GW*^ (polarization including electron screening and screened electron–hole interactions; Fig. 1a), *Σ*^*GW+Γ*^ (Fig. 1a, b), and *Σ*^*GW+Γ*+*Λ*^ (Fig. 1a–c). In the table, the first (second) number is the result using bare (dressed) Coulomb interactions in the ladders, and the third (our most sophisticated, in bold) is that using dressed interactions and energies. See also Fig. 2 for a graphical comparison of theory and experiment, and Extended Data Table 2 for more details.

### Benchmarking and general trends

We benchmark our approach against ECG (*ε*_b_ = 1,043 meV)^40^ and QMC (*ε*_b_ = 1,015 meV)^41^ calculations for LiH, and against QMC for formaldehyde (*ε*_b_ = 25 ± 3 meV)^42^. The LiH results demonstrate the general trends seen in all the molecules considered. The Hartree–Fock binding energy (*ε*_b_ = 130 meV) is severely deficient. Including the bare polarization attraction *Σ*^(2)^ considerably increases the binding energy (to *ε*_b_ = 434 meV). The addition of short-range screening corrections reduces the polarizability and binding energy (to *ε*_b_ = 336 meV, see Extended Data Table 2), but this is compensated by the inclusion of the electron–hole attractions (*Σ*^*GW*^: *ε*_b_ = 518 meV). This is still, however, less than half of the ECG result. The previous CI calculation^24^ is similarly deficient. Notably, however, including the virtual-Ps formation correlation potential (*Σ*^*GW+Γ*^) strongly enhances the binding, more than doubling it (to *ε*_b_ = 1,291 meV). Including the positron–hole ladder (*Σ*^*GW+Γ*+*Λ*^) slightly reduces binding (to *ε*_b_ = 1,106 meV); using screened interactions in the ladders reduces it slightly further (*ε*_b_ = 1,038 meV); additionally using the dressed energies in the diagram construction gives *ε*_b_ = 1,060 meV, agreeing with the ECG (QMC) result to within approximately 1% (approximately 4%). For formaldehyde, the addition of virtual-Ps again drastically enhances binding (by a factor of approximately 5 over the *GW* result), and including the positron–hole interaction results in a binding energy *ε*_b_ = 28 meV, within the error of the QMC calculation. Comparing to our method, the ECG and QMC approaches evidently account for virtual-Ps formation to a similar accuracy, although these methods cannot be scaled to larger molecules^40^, and provide relatively limited insight (see below). Additionally, the correlations effect a strong localization of the positron wavefunction density at the negatively charged end of the molecule (see Fig. 2), although overall, the wavefunction is quite diffuse, asymptotically taking the form *ψ* ∝ e^−*κr*^ where $\kappa =\sqrt{2{\varepsilon }_{{\rm{b}}}}$. We also calculate the positron Dyson wavefunction renormalization constants *a* (see equation (7) in Methods and Extended Data Table 2). These represent the contribution of the positron plus molecule in the ground-state component to the bound state. Their closeness to unity suggests the picture of a positron bound to the neutral molecule (instead of a Ps atom orbiting a molecular cation)^43^.

### Comparison with experiment and previous theory

**Extended Data Fig. 2:**
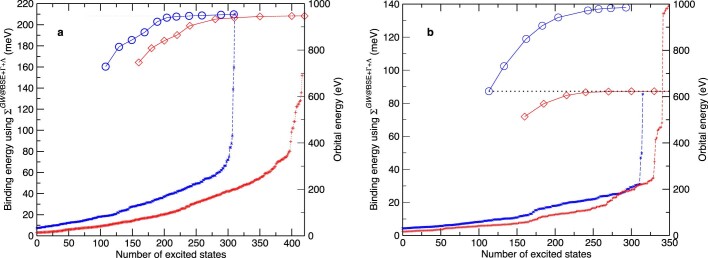
Convergence of positron binding energies in acetonitrile and CSe_2_ with respect to electron and positron basis size. Positron binding energy calculated using the *Σ*^*GW*@BSE+*Γ*+*Λ*^ self energy for varying number of electron (positron) Hartree–Fock molecular orbitals (whose energies are shown as blue and red crosses, respectively) included in the basis. For acetonitrile, the varying electron (positron) molecular orbital calculations included all positron (electron) molecular orbitals. For CSe_2_, the varying electron molecular orbital calculations included all positron molecular orbitals, whereas the varying positron molecular orbital calculation included 113 electron molecular orbitals (indicated by the lowest blue circle). The binding energy reaches convergence when the electronic orbital with energies up to ~150–200 eV are included. Similar behaviour was also observed for the other molecules considered. Source data

The best prior agreement between theory and experiment for any molecule was for acetonitrile (greater than approximately 25%). Considering the polar molecules first (Table 1 and Fig. 2), we immediately see that our full many-body theory (*Σ*^*GW+Γ*+*Λ*^) is superior, giving near-exact agreement (less than about 1% level) with experiment for propionitrile, propanal, acetaldehyde and formamide, and within 10% for acetonitrile and acetone (including the experimental error). (Overall we find excellent convergence in our calculation: see Methods and Extended Data Fig. 2). For all the polar molecules, the Hartree–Fock and bare (*Σ*^(2)^) and dressed (*GW*) polarization potentials substantially underestimate binding. The effect of virtual Ps is crucial: it enhances the binding energy by a factor of approximately 2 and is essential to bring theory into agreement with experiment. We note that the previous CI and APMO (‘REN-PP3’, which uses a diagonal approximation and does not explicitly account for virtual-Ps formation) calculations are severely deficient.

For the nonpolar molecules, we find that binding is exclusively enabled by correlations. For CS_2_ a considerable binding energy of 75 meV was measured, whereas the CI calculation failed to predict binding^27^. We see that polarization (*GW*) alone is insufficient to support binding. Notably, however, including the virtual-Ps contribution results in a prediction of large binding: our *Σ*^*GW+Γ*+*Λ*^ result of *ε*_b_ = 63 meV is close to experiment. For the nonpolar molecules the positron wavefunction is delocalized around the molecule (Fig. 2j–l), making the accurate description of virtual-Ps more computationally demanding. For CSe_2_ and benzene, in contrast to the molecules already considered, we have not optimized the bases (accurate calculation for these molecules require computational resources currently beyond our disposal) and our values for *ε*_b_ should be considered as lower bounds. Nevertheless, the results further elucidate the essential role of virtual-Ps formation in enabling (large) binding, and the positron wavefunctions provide fundamental insight that may prove instructive to refine ab initio and model calculations (see "Predicting binding in larger molecules: nucleobases").

### Prediction for formamide

For formamide, the archetypal molecule for the investigation of protein and peptide chemistry, we are unaware of any prior calculation. We predict binding (*ε*_b_ ≈ 189 meV). Preliminary experiments see evidence of *ε*_b_ ≈ 200 meV, although a final value has yet to be determined (J. R. Danielson, S. Ghosh & C. M. Surko, unpublished material).

### Molecular orbital contributions to binding

**Fig. 3:**
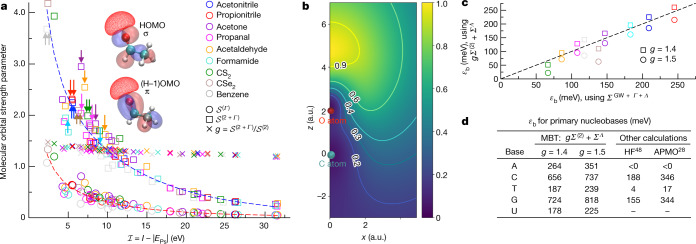
Molecular orbital contributions to binding, and scaling formula for large molecules. **a**, Molecular orbital contribution to the dimensionless strength of the virtual-Ps formation correlation potential ${{\mathscr{S}}}^{({\Gamma })}$ (circles) and including bare polarization ${{\mathscr{S}}}^{(2+{\Gamma })}$ (squares) and the ratio $g\equiv {{\mathscr{S}}}^{(2+{\Gamma })}/{{\mathscr{S}}}^{(2)}$ (crosses, where ${{\mathscr{S}}}^{(2)}={{\mathscr{S}}}^{(2+{\Gamma })}-{{\mathscr{S}}}^{({\Gamma })}$) against ${\mathcal I} \,=\,I\,-\,|{E}_{{\rm{Ps}}}|$, where *I* is the molecular orbital ionization energy and *E*_Ps_ = −6.8 eV is the ground-state energy of Ps. Arrows on ${{\mathscr{S}}}^{(2+{\Gamma })}$ mark π orbitals with *I* < 15 eV, and inset molecular orbital plots show HOMO (σ-type) and next HOMO (π-type) in acetaldehyde (solid red and blue: positive and negative regions of electronic molecular orbital; red wireframe: positron density at 85% of maximum). Dashed lines show the fits ${\mathscr{S}}\approx a{{\rm{e}}}^{-b {\mathcal I} }+c{ {\mathcal I} }^{-d}$ with *a* = 0.67 (2.57); *b* = 0.121 (0.092); *c* = 2.51 (6.64); *d* = 1.38 (1.37) for ${{\mathscr{S}}}^{(\Gamma )}$ (${{\mathscr{S}}}^{(2+{\Gamma })}$). **b**, The positron wavefunction density in acetaldehyde (in the plane containing the CO bond perpendicular to the CCO plane), which protrudes along the π bond. **c**, Comparison of binding energies for the molecules in **a** calculated using *Σ* = *Σ*^*GW*+*Γ*+*Λ*^ and accounting for (the computationally demanding to calculate) *Σ*^*Γ*^ via *Σ* = *gΣ*^(2)^ + *Σ*^*Λ*^, for *g* = 1.4 (circles) and 1.5 (squares) (see text). **d**, *ε*_b_ in nucleobases, calculated with *Σ* ≈ *gΣ*^(2)^ + *Σ*^*Λ*^ for *g* = 1.4 and *g* = 1.5. HF, Hartree–Fock; MBT, many-body theory. Source data

**Extended Data Fig. 3:**
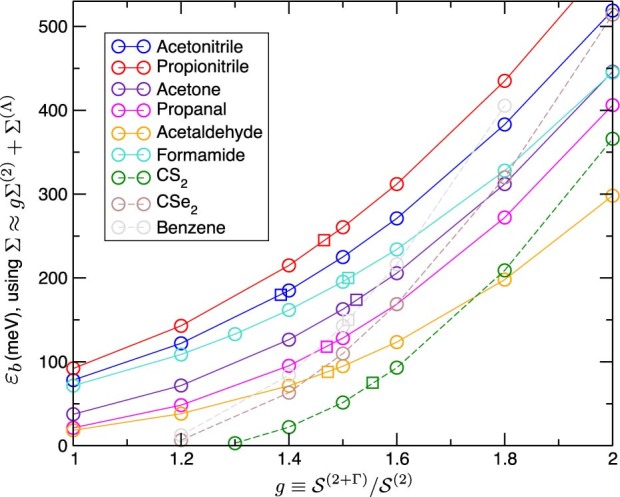
Nonlinearity of the binding energy and strength of correlation potential. Binding energy calculated approximating the positron self energy *Σ* as *Σ* ≈ *gΣ*^(2)^ + *Σ*^*Λ*^ as a function of the scaling parameter $g\equiv {{\mathscr{S}}}^{(2+\Gamma )}/{{\mathscr{S}}}^{(2)}$ (circles; see text for more details). Experiment (squares) is from refs. ^4,5^; for formamide preliminary measurements find a binding energy of *ε*_b_ ≈ 200 meV, but a final result is yet to be determined. See also Fig. 3c. Source data

At the static Hartree–Fock level, we find *ε*_b_ to be (monotonically and nonlinearly) related to the permanent dipole moment (expected from the dipole-potential model)^44^. Ultimately the correlation potential is anisotropic (see Extended Data Table 1 for calculated anisotropic polarizabilities), and depends nonlinearly on the polarizabilities and ionization energies of the individual molecular orbitals. Moreover, the binding energy depends nonlinearly on the correlation potential (for example, see Extended Data Fig. 3). The ordering of *ε*_b_ with respect to dipole moment persists to the *Σ*^(2*+Γ*+*Λ*)^ calculation, with the exception of acetaldehyde and propanal, and we note that for acetone, correlations considerably enhance *ε*_b_. It is instructive to consider the dimensionless quantity^45^ ${\mathscr{S}}=-{\sum }_{\nu  > 0}{\varepsilon }_{\nu }^{-1}\langle \nu |{\Sigma }|\nu \rangle$ (where the sum is over excited Hartree–Fock positron basis states of energy *ε*_*ν*_, see Methods), which gives an effective measure of the strength of the correlation potential *Σ*. The magnitudes of the strength of *Σ*^(2)^, ${{\mathscr{S}}}^{(2)}$ ranges from 4–15 (see Extended Data Table 2), and follows the ordering of the isotropic polarizability, with the exception of acetone and propanal (acetone has a larger polarizability and smaller ionization energy than propanal), and benzene and CSe_2_ (owing to benzene’s π bonds; see below). This suggests that (the short-range contributions to) *Σ*^(2)^ cannot be parametrized solely by the polarizability. Similarly, the magnitudes of ${{\mathscr{S}}}^{(\Gamma )}$ (ranging from 2–5) do not strictly follow the ordering of the ionization energies. To illuminate this, note that at the bare-polarization approximation, *Σ*^(2)^, and polarization plus virtual-Ps formation approximation, *Σ*^(2+*Γ*)^ = *Σ*^(2)^ + *Σ*^(*Γ*)^, we can delineate the contribution of individual molecular orbitals to positron binding. Figure 3a shows the partial ${{\mathscr{S}}}^{({\Gamma })}$ and ${{\mathscr{S}}}^{(2+{\Gamma })}$ for individual occupied molecular orbitals against their respective ionization energies, and the ratio $g\equiv {{\mathscr{S}}}^{(2+{\Gamma })}/{{\mathscr{S}}}^{(2)}$, where ${{\mathscr{S}}}^{(2)}={{\mathscr{S}}}^{(2+{\Gamma })}-{{\mathscr{S}}}^{({\Gamma })}$. Both ${{\mathscr{S}}}^{({\Gamma })}$ and ${{\mathscr{S}}}^{(2+{\Gamma })}$ decrease from the Ps-formation threshold to higher ionization energies: it is more difficult to perturb more tightly bound electrons. However, the decrease is not monotonic: we see that despite having larger ionization energies, π-type electronic molecular orbitals below the highest occupied molecular orbital (HOMO) can contribute considerably more than a σ-type HOMO to ${{\mathscr{S}}}^{({\Gamma })}$ and ${{\mathscr{S}}}^{(2+{\Gamma })}$—for example, in acetone, propanal and acetaldehyde, the strength of the π-type (H−1)OMO is larger than the σ-type HOMO, and in propanal, the (H−3)OMO of π type contributes more strongly than the (H−2)OMO, and so on. It was previously speculated^3^ that π bonds were important due to the ability of the positron to more easily access electron density that is delocalized from (repulsive) nuclei. This is borne out by our calculations, and we see in Fig. 3b that considerable positron density protrudes into the region of the π bond. Acetonitrile and propionitrile have a doubly degenerate π HOMO of large strength. For acetonitrile this results in a larger strength parameter than formamide.

### Predicting binding in larger molecules: nucleobases

The ratio $g\equiv {{\mathscr{S}}}^{(2+{\Gamma })}/{{\mathscr{S}}}^{(2)}$ depends weakly on the ionization energy, with a value of approximately 1.4–1.5 for the HOMOs (*I* ≈ 10 eV). We propose that binding energies of large molecules (for example, 15–100 atoms, for which a converged calculation of the virtual-Ps diagram (Fig. 1b) may be too computationally demanding) can be calculated by approximating *Σ* ≈ *gΣ*^(2)^ + *Σ*^*Λ*^. As well as accounting for virtual-Ps formation, this model potential reflects the anisotropy of the true interactions. For the molecules considered in Table 1, this works well (see Fig. 3c and Extended Data Fig. 3). Using this approximation, we calculate the positron binding energy in the five primary nucleobases (Fig. 3d). Our results are larger than the previous APMO calculations, mirroring the results for the molecules in Table 1. Notably, we predict binding in adenine.

### Positron lifetimes

**Extended Data Fig. 4:**
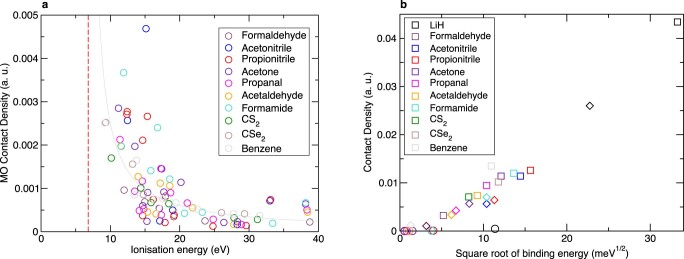
Calculated electron–positron contact density. **a**, Contact density for individual electronic molecular orbitals as a function of their ionization energy, calculated including vertex enhancement factors at *GW*@BSE+*Γ*+*Λ* level (see equation (6)). Red dashed line: positronium ground state energy at |*E*_Ps_| = 6.8 eV. Grey line: *δ*_ep_ = 0.008/(*I* − |*E*_Ps_|) (for a guide). Also see Extended Data Table 3 and Extended Data Fig. 5. **b**, Contact density calculated at the Hartree–Fock (circles) and various levels of many-body theory (diamonds: *GW*@BSE; squares: *GW*@BSE+*Γ*+*Λ*) against the square root of the binding energy. For nonpolar systems the *s*-type bound-state contact density is expected to show linear dependence (see equation 36 of the methods in ref. ^1^). For polar molecules there is no strict theoretical basis for this, although it is interesting that the data show an approximate linear dependency. Source data

**Extended Data Fig. 5:**
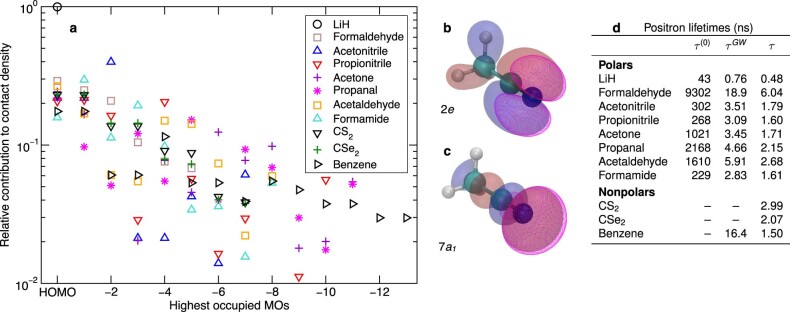
Calculated electron–positron contact densities and positron lifetimes with respect to annihilation. **a**, Fractional contribution of individual molecular orbitals to the total electron–positron contact density (equation (6)). **b**, **c**, The electron–positron contact density (magenta) at the *Σ*^*GW+Γ*+*Λ*^ level for the (doubly degenerate) HOMO and (H−2)OMO in acetonitrile (blue and brown show negative and positive electron wavefunction regions, respectively), compare with Fig. 2c (positron density). **d**, Positron lifetimes with respect to annihilation. *τ*^(0)^: lifetime calculated in the Hartree–Fock independent particle approximation excluding the vertex enhancement factors and using a positron wavefunction normalized to unity; *τ*^*GW*^ and *τ*: lifetime calculated using the Dyson positron wavefunction at the *Σ*^*GW*^ and *Σ*^*GW+Γ*+*Λ*^ levels including vertex enhancement factors and renormalization constants (equations (6), (7)). Source data

We also calculate the annihilation lifetime of the bound positron (see Methods and Extended Data Figs. 4, 5), finding that the correlations reduce it by approximately 2–3 orders of magnitude to *τ* ≈ 1 ns. The partial annihilation rates on individual occupied electron orbitals are also calculated and are shown to depend strongly on the symmetry relative to that of the positron molecular orbital, with the HOMO not necessarily dominating, for example in acetonitrile and formamide. Such partial annihilation rates are required to properly interpret materials science experiments—for example, positron-annihilation-induced Auger electron spectroscopy^7,8^—and define the initial cationic wavepackets in positron-annihilation-induced charge migration^46^, relevant to, for example, base-selective oxidization of nucleobases^47^.

## Future perspectives

Many-body theory has elucidated the long-standing correlation-dominated problem of positron binding to molecules. Benchmarking against ECG and QMC calculations for LiH and formaldehyde showed the many-body theory to be similarly accurate, but its power is that it can be extended to large molecules and provides additional fundamental insight. Importantly, the effects of correlations were quantified and delineated. In particular, the key role of virtual-Ps formation in greatly enhancing binding in polar molecules and exclusively enabling binding in nonpolar molecules, the near-cancellation of screening corrections to the bare polarization, and the non-negligible role of the positron–hole interaction were all identified. The contribution of individual molecular orbitals to the (anisotropic) correlation potential was quantified, and the importance of electronic π orbitals (previously speculated)^3^ was confirmed. For polar molecules the many-body theory gave binding energies in excellent (near exact, or within small error bars for most cases) agreement with the long-standing experiments. For nonpolar molecules, binding was predicted for CS_2_, CSe_2_ and benzene, with larger discrepancy (within less than approximately 20%) owing to slower convergence of the virtual-Ps contribution due to the delocalized positron wavefunction. We also predicted binding in formamide and the primary nucleobases. The excellent level of agreement with experiment reaffirms that for these molecules binding is dominated by correlations, and that vibrational effects are relatively small^1,28–32^. Complementary laser-assisted photorecombination experiments^48^ would provide direct comparison with our results, elucidating the problem in the absence of vibrations. Coupled-cluster^34^ and ADC^36^ calculations should also be possible and instructive.

The present calculations support resonant annihilation experiments and the related theory to which binding energies and annihilation lifetimes enter as parameters^1^. Beyond the fundamental insight immediately provided, the step-change in capability enables calculations of positron scattering and molecular-orbital-resolved, Doppler-broadened annihilation γ-ray spectra (underway), required to, for example, properly interpret positron-based ultrasensitive materials science techniques^7,8^, provide insight on molecular fragmentation^10^, and understand positron interactions in the Galaxy^11^ and living tissue (relevant to developing next-generation PET)^49,50^. It also provides a foundation for the implementation of inelastic^38^ (to include real Ps formation) and time-dependent^51^ molecular processes, to, for example, model positron-annihilation-induced Auger-electron spectroscopy^8^, interatomic Coulomb decay^52^, charge migration^46^ (including that relevant to medicine)^47^ and luminescence^53^, and for the study of vibrational effects via coupling of the nuclear and electronic degrees of freedom^54^. Finally, the difficulty of the positron–molecule problem presents a rich testbed for the development of other approaches to the many-body problem, for which our results can serve as benchmarks.

## Methods

### The Dyson equation in a Gaussian basis

We calculate the positron–molecule binding energy *ε* and quasiparticle wavefunction *ψ*_*ε*_ by solving the Dyson equation. We take the zeroth-order Hamiltonian *H*^(0)^ to be that of the positron in the Hartree–Fock field of the frozen-target *N*-electron ground-state molecule. The self-energy diagrams thus begin at second order in the Coulomb interaction. Instead of computing the self energy *Σ*(**r**,**r**′) in the coordinate basis, it is more convenient to work with its matrix elements in the Hartree–Fock basis. Specifically, we expand the electron (−) and positron (+) Hartree–Fock molecular orbitals ${\phi }_{a}^{\pm }({\bf{r}})$ in distinct Gaussian basis sets as ${\phi }_{a}^{\pm }({\bf{r}})={\sum }_{A}^{{N}_{c}^{\pm }}{\sum }_{k=1}^{{N}_{A}^{\pm }}{C}_{aAk}^{\pm }{\chi }_{{A}_{k}}^{\pm }({\bf{r}})$, where *A* labels the ${N}_{c}^{\pm }$ basis centres, *k* labels the ${N}_{A}^{\pm }$ different Gaussians on centre *A*, each taken to be of Cartesian type with angular momentum *l*^*x*^ + *l*^*y*^ + *l*^*z*^, and with ${\chi }_{{A}_{k}}({\bf{r}})={{\mathscr{N}}}_{{A}_{k}}{(x-{x}_{A})}^{{l}_{Ak}^{x}}{(y-{y}_{A})}^{{l}_{Ak}^{y}}{(z-{z}_{A})}^{{l}_{Ak}^{z}}\exp \{-{\zeta }_{Ak}|{\bf{r}}-{{\bf{r}}}_{A}{|}^{2}\}$, where ${{\mathscr{N}}}_{{A}_{k}}$ is a normalization constant, and *C* are the expansion coefficients to be determined (see later in this section). Molecular geometries are determined via minimization of the total electronic Hartree–Fock energy in the Molpro^56,57^ package, for the respective basis set (see next paragraph), ensuring an internally consistent ab initio calculation.

For both electrons and positrons, we use the diffuse-function-augmented correlation-consistent polarized aug-cc-pVXZ (X = T or Q) Dunning basis sets centred on all atomic nuclei of the molecule, which enables accurate determination of the electronic structure including cusps^58^ and expulsion of the positron density from the nuclei. To capture the long-range correlation effects, for the positron we also additionally include at least one large even-tempered set at the molecular centre or region of maximum positron density of the form *Ns*(*N* − 1)*p*(*N* − 2)*d*(*N* − 3)*f*(*N* − 4)*g* with *N* ≈ 10–15 (where it should be understood that the full degenerate set of nonzero angular momentum functions is used) and exponents ${\zeta }_{{A}_{k}}={\zeta }_{{A}_{1}}{\beta }^{k-1}$, *k* = 1, …, *N*, for each angular momentum type, where ${\zeta }_{{A}_{1}} > 0$ and *β* > 1 are parameters. The value of ${\zeta }_{{A}_{1}}$ is important because the bound positron wavefunction behaves asymptotically as *ψ* ∝ e^−*κr*^, where $\kappa =\sqrt{2{\varepsilon }_{{\rm{b}}}}$. Thus, to ensure that the expansion describes the wavefunction well at *r* ≈ 1/*κ*—that is, that the broadest Gaussian covers the extent of the positron wavefunction—one must have ${\zeta }_{{A}_{1}}\lesssim {\kappa }^{2}=2{\varepsilon }_{{\rm{b}}}$. In practice we performed binding energy calculations for a range of ${\zeta }_{{A}_{1}}$ and *β* for each molecule, finding that there are broad ranges of stability. The optimal ${\zeta }_{{A}_{1}}$ was typically found to be in the range of 10^−4^–10^−3^ for *s*- and *p*-type Gaussians and 10^−3^–10^−2^ for *d*- and *f*-type Gaussians, whereas *g*-type Gaussian exponents usually had ${\zeta }_{{A}_{1}}={10}^{-1}$ (atomic units are assumed throughout unless otherwise specified). The optimal *β* ranges from 2.2 to 3.0 depending on the number of functions *N* in a given shell. Finally, to simultaneously describe the expulsion of the positron wavefunction from the nuclei, and accurately describe positron density maxima away from the molecule, we strategically place additional (H atom) aug-cc-pVXZ (X = T, Q) basis sets on ‘ghost’ centres close to the regions of maximum positron density. These additional Gaussians provide additional flexibility in the basis, enabling a better description of the positron wavefunction. In particular, they play an important role in enabling the accurate description of virtual-Ps formation, which occurs several atomic units away from the molecule, and which would require high angular momentum functions to resolve the electron–positron distance in Ps away if a single basis centre was used^19^. By placing Gaussian basis functions of angular momentum (we use *l* ≤ 4) on multiple ghost centres, higher angular momentum functions can be effectively generated in the interstitial regions (see appendix B of ref. ^59^ for details). In practice, for each molecule, we perform calculations with successively increasing number of ghost centres, whose locations are optimized manually until the binding energy stabilizes . The optimum locations are found iteratively: the calculation with zero ghosts generally indicates the region of maximum positron density, around which the ghosts are targeted in subsequent calculations, thus improving the density. As a general rule, we found that the optimal distance of the ghost centres from the atom closest to the maximum of positron density is about 1 Å. For some larger or nonpolar molecules, we use multiple ghost centres surrounding the molecule. To check convergence with respect to the number and location of these ghost centres, for each molecule we performed calculations including TZ or QZ bases on a successively increasing number of ghost centres in different arrangements until the increase in binding energy fell below a few per cent. We found that including ghosts can increase binding energies by ~10% in the polar molecules, and easily by ~30% for the nonpolar ones—for example, for CS_2_ we obtained *ε*_b_ = 39 meV at *GW*@BSE+*Γ*+*Λ* level with no ghosts, rising to *ε*_b_ = 68 meV with 16 additional ghosts. The use of higher angular momenta and more ghosts could be expected to further increase the binding energies of the nonpolar molecules. We also investigated the difference of using aug-cc-pVXZ for X = T, Q in the atomic-centred and ghost bases, and higher angular momenta in the even-tempered basis. Some improvement was noted moving from X = T to Q, and also when *g* states were included in addition to *f*, to a level of a 5%–10% in polar molecules, and 10%–30% in nonpolar molecules. Overall, good convergence with respect to both the electron and positron bases was observed (see for example, Extended Data Fig. 2).

The coefficients *C* in the expansion of the positron wavefunction in Gaussians are found by solving the Roothaan equations *F*^±^*C*^±^ = *S*^±^*C*^±^*ε*^±^, where *F*^±^ is the Fock matrix and *S* is the overlap matrix. The one-body and two-body Coulomb integrals of the Fock matrix are calculated using the McMurchie–Davidson algorithm^60^. We eliminate linearly dependent states by excluding eigenvalues <10^−5^ of the overlap matrices (typically ≲5% of the states). In practice, to minimize the basis dimensions we transform all quantities to a spherical harmonic Gaussian basis (for a given angular momentum, the number of Cartesian Gaussians is greater than or equal to the number of spherical harmonic Gaussians)^61^. Solution of the Roothaan equations yield bases of electron and positron Hartree–Fock molecular orbitals $\{{\phi }_{\alpha }^{\pm }({\bf{r}})\}$ (which include ground and other negative energy states, and discretized continuum states) with which the self-energy diagrams can be constructed (see the next section for details).

Expanding the positron Dyson wavefunction in the positron Hartree–Fock molecular orbital basis as ${\psi }_{\varepsilon }({\bf{r}})={\sum }_{\nu }{D}_{{\rm{\nu }}}^{\varepsilon }{\phi }_{\nu }^{+}({\bf{r}})$ transforms the Dyson equation to the linear matrix equation *HD* = *εD*, where $\langle {\nu }_{1}|H|{\nu }_{2}\rangle ={\varepsilon }_{{\nu }_{1}}{\delta }_{{\nu }_{1}{\nu }_{2}}+\langle {\nu }_{1}|{{\Sigma }}_{\varepsilon }|{\nu }_{2}\rangle$. Note that we calculate the full self-energy matrix including off-diagonal terms. Such a non-perturbative approach is essential for nonpolar molecules, where binding is enabled exclusively by correlations. In practice, to obtain the self-consistent solution to the Dyson equation, we calculate the self energy at a number of distinct energies *E*_*i*_ spanning the true binding energy *ε*_b_, with the latter determined from the intersection of the *ε*_b_(*E*_*i*_) data with the line *ε*_b_(*E*) = *E*.

### The positron–molecule self energy

As discussed in the main text (Fig. 1), we consider three contributions to the irreducible self energy of the positron in the field of the molecule: *Σ*^*GW*^ (which describes polarization, screening and electron–hole interactions); *Σ*^*Γ*^ (which describes the non-perturbative process of virtual-Ps formation); and *Σ*^*Λ*^ (which includes the infinite ladder series of positron–hole interactions). In practice, we construct the individual contributions by first solving the respective Bethe–Salpeter equations (BSE; see Extended Data Fig. 1) for the electron–hole polarization propagator *Π*, the two-particle positron–electron propagator ${G}_{{\rm{II}}}^{{\rm{ep}}}$ and the positron–hole two-‘particle’ propagator^33^ ${G}_{{\rm{II}}}^{{\rm{ph}}}$. Their general form is *L*(*ω*) = *L*^(0)^(*ω*) + *L*^(0)^(*ω*)*KL*(*ω*), where the *L*^(0)^ are non-interacting two-body propagators and *K* are the interaction kernels^33,62,63^ (for example, see Extended Data Fig. 1e for the BSE for the electron–hole polarization propagator *Π*). In the excitation space of pair product Hartree–Fock orbitals *L* = (*Cω* − *H*)^−1^ = *ξ*(*ω* − *Ω*)^−1^*ξ*^−1^*C* ^−1^, where the pair transition amplitudes *ξ* are the solutions of the pseudo-Hermitian linear-response generalized eigenvalue equations^63–65^ *Hξ* = *CξΩ*, *ξ* ^†^*Cξ* = *C*, where

1 $$H=(\begin{array}{cc}A & B\\ {B}^{\ast } & {A}^{\ast }\end{array});\,\xi =(\begin{array}{cc}X & {Y}^{\ast }\\ Y & {X}^{\ast }\end{array});\,C=(\begin{array}{cc}1 & 0\\ 0 & -1\end{array});\,\Omega \,=\,(\begin{array}{cc}{\Omega }_{+} & 0\\ 0 & {\Omega }_{-}\end{array}),$$

for excitation energies ${{\Omega }}_{+}^{\alpha }$ and ${{\Omega }}_{-}^{\alpha }$, which are labelled by *α* = 1, …, dim(*A*). Here the *A* and *B* matrices depend on the particular two-particle propagator *L* under consideration and the approximation used for it (see Extended Data Table 4 for the explicit matrix elements): note that *B* = 0 for the two-particle propagators involving the positron, because the vacuum state for the diagrammatic expansion is that of the *N*-electron molecule, and thus there are no positron holes and only time-forward positron propagators. To determine the amplitudes, we use the parallel diagonalization algorithm of a previous work^66^, which exploits a similarity transform that gives the eigenvalues of *C*^−1^*H* as the square roots of the eigenvalues of (*A* + *B*)(*A* − *B*) (thus requiring matrices of dimension of the *A* block, that is, half of the full BSE matrix dimension) to obtain $X=\frac{1}{2}({L}_{2}U+{L}_{1}V){{\Omega }}_{+}^{-1/2}$ and $Y=\frac{1}{2}({L}_{2}U-{L}_{1}V){{\Omega }}_{+}^{-1/2}$, via the Cholesky decompositions $A+B={L}_{1}{L}_{1}^{{\rm{T}}}$ and $A-B={L}_{2}{L}_{2}^{{\rm{T}}}$, and the singular value decomposition ${L}_{2}{L}_{1}^{{\rm{T}}}=U{\Omega }{V}^{{\rm{T}}}$, where ^T^ indicates the transpose. The positron–molecule self-energy matrix elements can then be written as:

2 $$\langle {\nu }_{1}|{\Sigma }_{E}^{GW}|{\nu }_{2}\rangle =\sum _{\alpha ,{\nu }_{3}}\frac{{w}_{{\nu }_{1}{\nu }_{3}}^{\Pi ,\alpha }{w}_{{\nu }_{2}{\nu }_{3}}^{\Pi ,\alpha }}{E-{{\rm{\varepsilon }}}_{{\nu }_{3}}-{\Omega }_{+,\alpha }^{\Pi }+{\rm{i}}\eta },$$

3 $$\langle {\nu }_{1}|{\Sigma }_{E}^{\Gamma }|{\nu }_{2}\rangle =\sum _{\alpha ,n}\frac{{w}_{{\nu }_{1}n}^{\Gamma ,\alpha }{w}_{{\nu }_{2}n}^{\Gamma ,\alpha }}{E-{\Omega }_{\alpha }^{\Gamma }+{{\rm{\varepsilon }}}_{n}+{\rm{i}}\eta }-\langle {\nu }_{1}|{\Sigma }_{E}^{(2)}|{\nu }_{2}\rangle ,$$

4 $$\langle {\nu }_{1}|{\Sigma }_{E}^{\Lambda }|{\nu }_{2}\rangle =\sum _{\alpha ,\mu }\frac{{w}_{{\nu }_{1}\,\mu }^{\Lambda ,\alpha }{w}_{{\nu }_{2}\,\mu }^{\Lambda ,\alpha }}{E-{\Omega }_{\alpha }^{\Lambda }-{{\rm{\varepsilon }}}_{\mu }+{\rm{i}}\eta }-\langle {\nu }_{1}|{\Sigma }_{E}^{(2)}|{\nu }_{2}\rangle ,$$

where *ν*_1_, *ν*_2_ and *ν*_3_ denote positron indices and *μ* and *n* denote electron excited states and holes respectively, *Σ*^(2)^—which results from the *Π*^(0)^ contribution to *Σ*^*GW*^ and is present in both ${G}_{{\rm{II}}}^{{\rm{ep}}}$ and ${G}_{{\rm{II}}}^{{\rm{ph}}}$—is subtracted to prevent double counting, and

5 $$\begin{array}{c}{w}_{{\nu }_{1}{\nu }_{3}}^{\Pi ,\alpha }=\sum _{\mu n}({\nu }_{1}{\nu }_{3}|\mu n)({X}_{\mu n}^{\Pi ,\alpha }+{Y}_{\mu n}^{\Pi ,\alpha }),\\ {w}_{{\nu }_{1}n}^{\Gamma ,\alpha }=\sum _{\mu {\nu }_{3}}({\nu }_{1}n|{\nu }_{3}\mu ){X}_{{\nu }_{3}\mu }^{\Gamma ,\alpha },\\ {w}_{{\nu }_{1}\mu }^{\Lambda ,\alpha }=\sum _{n{\nu }_{3}}({\nu }_{1}\mu |{\nu }_{3}n){X}_{{\nu }_{3}n}^{\Lambda ,\alpha },\end{array}$$

where chemists’ notation for Coulomb matrix elements (*ν*_1_*ν*_3_|*μn*) and so on is used (see Extended Data Table 4). The total self energy is calculated as *Σ* = *Σ*^*GW*^ + *Σ*^*Γ*^ + *Σ*^*Λ*^. Such addition of the individual channels is routine in atomic many-body theory calculations^19,67,68^ and in condensed matter, for example, the fluctuation-exchange (‘FLEX’) approximation^69–71^. We note that the above approach is restricted to molecules with ionization energies larger than the ground-state energy of Ps (6.8 eV). For these, the Ps-formation channel is closed, and Ps formation proceeds as a virtual process (with the electron temporarily tunnelling to the positron). For molecules for which the ionization is smaller than the energy of ground-state Ps, the inelastic Ps-formation channel is open. The above approach does not account for such inelastic channels. We note, however, that there are methods proposed to include inelastic channels in a many-body formalism^38^. Its implementation is beyond the scope of this paper, but would be a worthwhile future endeavour.

We implement the above in the massively parallelized EXCITON+ code developed by us, adapting the EXCITON code^72–74^ to include positrons. EXCITON employs density-fitting (of the electronic density) methods^74–79^ in a Gaussian-orbital basis for calculation of the electronic self-energy and four-centre integrals that appear in the *A* and *B* matrices of the BSE for finite^73^ and periodic^72,74^ systems. The EXCITON+ code developed at Queen’s University Belfast adapts EXCITON to additionally solve the positron–molecule Hartree–Fock problem, construct the full (nondiagonal) positron–molecule self energy (calculating *w*^*Π*^, *w*^*Γ*^ and *w*^*Λ*^ via density fitting of the electronic density, and including screening terms in the ladders), and solve the Dyson equation and calculate the positron–electron contact density (lifetime with respect to annihilation). The use of density fitting reduces four-centre Coulomb integrals to products of three-centre Coulomb integrals and matrix elements of the Coulomb operator between atomic orbital basis functions. Thus, the memory scaling is approximately ${N}_{-}^{2}{M}_{-}$, where ${N}_{-}$ is the total number of electron basis functions, and ${M}_{-}\gtrsim 3{N}_{-}$ is the number of electron auxiliary basis functions. The most computationally demanding part of our approach is in the calculation of the virtual-Ps self-energy contribution *Σ*^*Γ*^. For this, dim*A* = dim*X*^*Γ*^ = *N*_*ν*_ × *N*_*μ*_, the product of total number of positron molecular orbitals and excited electron molecular orbitals. For the calculations considered here, *N*_*ν*_ ranged from 400–500 and *N*_*μ*_ from 300–400, resulting in dim*X*^*Γ*^ = 120,000–200,000; thus, diagonalizing the matrix of (dim*X*^*Γ*^)^2^ elements demanded between ~100 GB and 1.5 TB of random access memory (RAM). The calculations were performed on two AMD EPYC 128 CPU @ 2 GHz, 768 GB RAM nodes of the United Kingdom Tier-2 supercomputer ‘Kelvin-2’ at Queen’s University Belfast. By contrast, the *GW* calculations involve dim*A* = dim*X*^*Π*^ ≤ *N*_*ν*_ × *N*_*n*_, that is, a maximum equal to the product of the number of occupied and excited electron molecular orbitals. In practice, not all occupied orbitals need to be included because the tightly bound lowest occupied molecular orbitals (LOMOs) are less susceptible to perturbation by the positron and have negligible contribution to the self energy. Thus, because *N*_*n*_ ≪ *N*_*μ*_ < *N*_*ν*_, ab initio *GW*@RPA/TDHF/BSE calculations (RPA, random phase approximation; TDHF, time-dependent Hartree–Fock; BSE, Bethe–Salpeter equation) are considerably less computationally expensive, and can be performed for molecules or clusters with ~100 atoms, providing at least lower bounds on the positron binding energies. Moreover, as discussed (see Fig. 3c and Extended Data Fig. 3) and demonstrated for nucleobases (Fig. 3d), the virtual-Ps formation contribution can be approximated by scaling the *Σ*^(2)^ self energy by the strength parameter ratio $g\equiv {{\mathscr{S}}}^{(2+{\Gamma })}/{{\mathscr{S}}}^{(2)}$, namely *Σ* ≈ *gΣ*^(2)^ + *Σ*^*Λ*^, thus enabling computationally relatively inexpensive binding-energy calculations that account for virtual-Ps formation for molecules of ~100 atoms. Ab initio calculations for larger molecules including the virtual-Ps self energy will be feasible with additional computational resources, as would calculations using different truncated product spaces of excited electron and positron molecular orbitals and extrapolating to the basis set limit.

### Improving the accuracy of calculations

As mentioned in the previous section, the computationally intensive calculations presented here were performed using relatively modest computational resources. Access to national supercomputing facilities would enable more complete basis sets and further exploration of the effect of ghost basis centres. Numerical accuracy can also be systematically improved in a number of ways. Exploiting the molecular point group symmetry via symmetry-adapted bases and using integral screening techniques would improve the efficiency of the calculations, enabling more complete basis sets to be used. This would ultimately improve the description of the correlations (particularly in generating higher angular momenta for improved description of the virtual-Ps formation process). The calculation of the positron–molecule self energy can be improved by implementing a self-consistent diagram approach in which the positron–molecule self energy is built from *GW* calculated electron and positron Dyson orbitals instead of Hartree–Fock ones^33,80^, and/or by coupling the three self-energy channels *Σ*^*GW*^, *Σ*^*Γ*^ and *Σ*^*Λ*^ by approximating the three-particle propagators via the Faddeev^81^, parquet^69^ or ADC(3)^36^ methods (expected to be computationally feasible for small molecules using national supercomputing facilities). Moreover, the diagrammatic series should be amenable to a diagrammatic Monte Carlo^82,83^ prescription, a powerful stochastic simulation method that enables the effective summation of many more (classes of) diagrams than considered here.

### Positron annihilation rate in the bound state

The solution of the Dyson equation also yields the positron bound-state wavefunction *ψ*_*ε*_. Using it, the 2*γ* annihilation rate in the bound state ${\Gamma }={\rm{\pi }}{r}_{0}^{2}c{\delta }_{{\rm{ep}}}$ (*Γ* [ns^−1^] = 50.47*δ*_ep_ [a.u.])—the inverse of which is the lifetime of the positron–molecule complex with respect to annihilation—can be calculated. Here *r*_0_ is the classical electron radius, *c* is the speed of light and *δ*_ep_ is the electron–positron contact density,

6 $${\delta }_{{\rm{e}}{\rm{p}}}=\mathop{\sum }\limits_{n=1}^{{N}_{{\rm{e}}}}{\gamma }_{n}\int |{{\rm{\phi }}}_{n}({\bf{r}}){|}^{2}|{\psi }_{{\rm{\varepsilon }}}({\bf{r}}){|}^{2}{\rm{d}}{\bf{r}},$$

**Extended Data Fig. 6:**
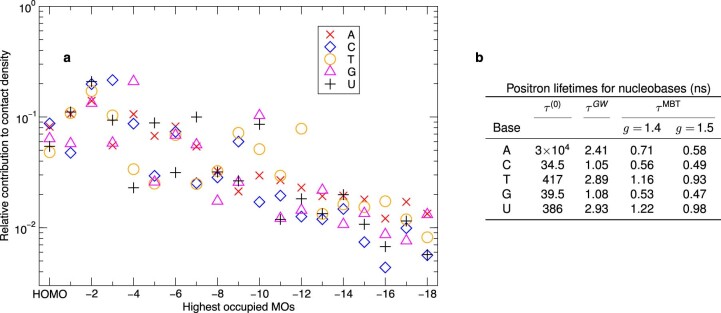
Calculated electron–positron contact densities and positron lifetimes with respect to annihilation for nucleobases. **a**, Fractional contribution of individual molecular orbitals to the total electron–positron contact density (equation (6)). **b**, Table of positron lifetimes with respect to annihilation. *τ*^(0)^: lifetime calculated in the Hartree–Fock independent particle approximation excluding the vertex enhancement factors and using a positron wavefunction normalized to unity; *τ*^*GW*^ and *τ*^MBT^: lifetime calculated using the Dyson positron wavefunction at the *Σ*^*GW*^ and *gΣ*^(2)^ + *Σ*^(*Λ*)^ levels for *g* = 1.4 and 1.5 including vertex enhancement factors and renormalization constants (equations (6), (7)). Source data

Here the sum is over all *N*_e_ occupied electron molecular orbitals with wavefunctions *φ*_*n*_, and *γ*_*n*_ are molecular-orbital-dependent enhancement factors that account for the short-range electron–positron attraction^20,84^. Recent many-body calculations for atoms by one of us determined them to follow a physically motivated scaling with the ionization energy^20,84^ ${\gamma }_{n}=1+\sqrt{1.31/|{\varepsilon }_{n}|}+{(0.834/|{\varepsilon }_{n}|)}^{2.15}$ (where quantities are in a.u.), which we assume to hold here. The positron Dyson wavefunction is a quasiparticle wavefunction that is the overlap of the wavefunction of the *N*-electron ground state molecule with the fully correlated wavefunction of the positron plus *N*-electron molecule system^33^. It is normalized as

7 $$\int |{\psi }_{\varepsilon }({\bf{r}}){|}^{2}\,{\rm{d}}{\bf{r}}={(1-\partial \varepsilon /\partial E{|}_{{\varepsilon }_{{\rm{b}}}})}^{-1}\equiv a < 1,$$

which estimates the contribution of the ‘positron plus molecule in the ground state’ component to the positron–molecule bound-state wavefunction, that is, the degree to which the positron–molecule bound state is a single-particle state, with smaller values of *a* signifying a more strongly correlated state. Extended Data Figs. 4, 5 present contact density data. Extended Data Fig. 4a shows the individual molecular orbit contribution to the contact density as a function of the molecular orbit ionization energy. As in Fig. 3 (contribution of strength parameters from individual molecular orbits), overall the contact density increases as the ionization energy decreases: the positron overlap is greater with the more diffuse electronic HOMOs. However, molecular orbitals below the HOMO can in fact dominate, for example, acetonitrile, as shown further in Extended Data Fig. 5a–c, and Extended Data Fig. 6 for the primary nucleobases.

## Online content

Any methods, additional references, Nature Research reporting summaries, source data, extended data, supplementary information, acknowledgements, peer review information; details of author contributions and competing interests; and statements of data and code availability are available at 10.1038/s41586-022-04703-3.

### Source data

Source Data Fig. 2

Source Data Fig. 3

Source Data Extended Data Fig. 2

Source Data Extended Data Fig. 3

Source Data Extended Data Fig. 4

Source Data Extended Data Fig. 5

Source Data Extended Data Fig. 6

## Figures and Tables

**Extended Data Table 1:**
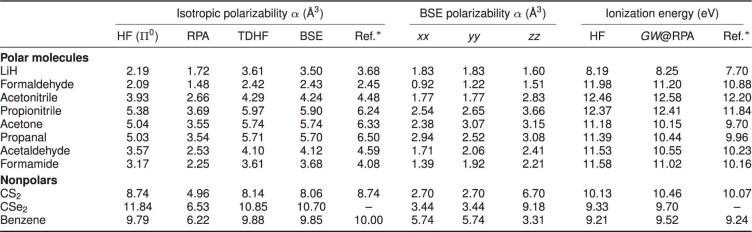
Calculated polarizabilities (in Å^3^) and ionization energies (in eV)

^*^Reference values from ref. ^85^.

Molecules are oriented such that the main axis of symmetry, or the main bond (C–O, C–N), above which the positron density is localized, is along *z*. The isotropic value is given by a sum of *xx*, *yy*, and *zz* terms multiplied by 2/3. Note that the *zz* components have larger differences between molecules than isotropic polarizabilities, for example, for propionitrile, acetone and propanal the isotropic polarizabilities are within 1% of each other, whereas the *zz* components differ by ~15%. Ionization energies calculated at the *GW*@RPA level were performed using the diagonal approximation for the electron–molecule self energy *Σ*^(−)^, that is, ${\tilde{\varepsilon }}_{\mu }={\varepsilon }_{\mu }+Z\langle \mu |{{\Sigma }}_{{\varepsilon }_{\mu }}^{(-)}|\mu \rangle$, where $Z\equiv {(1-\partial {{\Sigma }}_{E}/\partial E)}^{-1}{|}_{{\varepsilon }_{\mu }}$ (ref. ^69^). HF, Hartree–Fock; RPA, random phase approximation; TDHF, time-dependent Hartree–Fock; BSE, Bethe–Salpeter equation.

**Extended Data Table 2:**
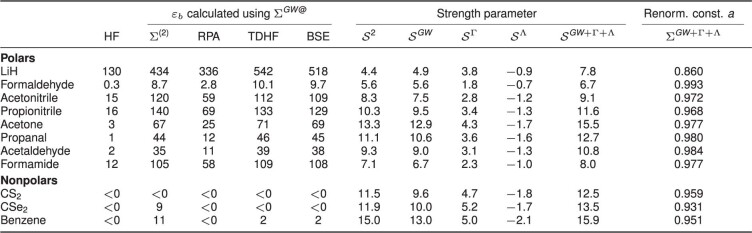
Positron binding energies in the *GW* approximation (meV), dimensionless correlation-potential strength parameters and Dyson wavefunction renormalization constants *a*

Positron binding energies (in meV, complementary data to Table 1) calculated at the Hartree–Fock and various levels of the *GW* approximation (see Extended Data Fig. 1): *Σ*^(2)^ (bare polarization propagator); RPA (random phase approximation); TDHF (time-dependent Hartree–Fock approximation); and BSE (Bethe–Salpeter equation). Dimensionless strength parameter of the correlation potential (defined in the text) in different approximations to the positron–molecule self energy (see Fig. 3). Positive (negative) strength parameters denote attractive (repulsive) positron–molecule interactions. The final column gives the calculated positron Dyson wavefunction renormalization constants *a* for the *Σ*^*GW+Γ*+*Λ*^ calculation (see equation (7)).

**Extended Data Table 3:**
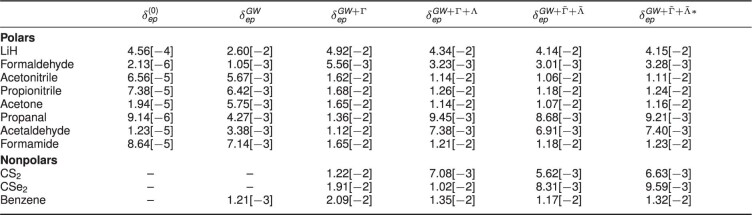
Positron–molecule annihilation contact densities (a.u.)

Electron–positron contact densities calculated from equation (6) including molecular-orbital-dependent enhancement factors and Dyson orbital renormalization constants *a* (equation (7)), in different approximations to the positron Dyson wavefunction: ${\delta }_{{\rm{ep}}}^{(0)}$, using Hartree–Fock positron wavefunction; ${\delta }_{{\rm{ep}}}^{GW}$, using the Dyson wavefunction calculated with the *GW*@BSE self energy; ${\delta }_{{\rm{ep}}}^{GW+{\Gamma }}$, using the Dyson wavefunction calculated with the *GW*@BSE plus virtual-Ps self energy; ${\delta }_{{\rm{ep}}}^{GW+\varGamma +\varLambda }$, using the Dyson wavefunction calculated with the *GW*@BSE plus virtual Ps plus positron–hole self energy with unscreened Coulomb interactions in the *Γ* and *Λ* ladders. Numbers in brackets indicate powers of 10; hyphens denote approximations in which the positron does not bind.

**Extended Data Table 4:**
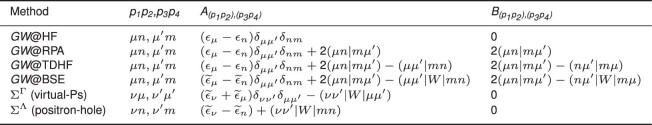
Matrix elements of the Bethe–Salpeter linear response Hamiltonian

Elements of the *A* and *B* blocks of the linear-response Hamiltonian matrices that result from the BSE equations (equation (1)) for the electron–hole propagator, the positron–electron propagator, and the positron–hole propagator. Chemists’ notation for Coulomb matrix elements in the molecular orbital basis is used, $(\nu \mu |\nu {\prime} \mu {\prime} )=\int {\rm{d}}{\bf{r}}{\rm{d}}{\bf{r}}{\prime} {\phi }_{\nu }^{\ast }({\bf{r}}){\phi }_{\mu }({\bf{r}})v({\bf{r}},{\bf{r}}{\prime} ){\phi }_{{\mu }^{{\prime} }}^{\ast }({\bf{r}}{\prime} ){\phi }_{{\nu }^{{\prime} }}({\bf{r}}{\prime} )$, where (*μ* and *μ*′), (*n* and *m*) and (*ν* and *ν*′) denote electron particles, electron holes and positron particles, respectively. Factors of two arise from summation over spin, and tildes on energy eigenvalues for BSE denote that these are calculated at the level of *GW*@RPA. For the virtual-Ps and positron–hole matrices, *B* = 0 because there are no positron ‘holes’ in the *N*-electron ground-state molecule vacuum state, and thus only time-forward diagrams are present in the positron single-particle propagator and two-particle propagators (that is, here the ‘Tamm–Dancoff approximation’ is exact for positrons). Matrix elements of the dressed Coulomb interaction *W* = *v* + *W*_d_ (Extended Data Fig. 1b), where *W*_d_ = *vΠ*^RPA^*v* is the dynamic part determined from the polarization propagator in the random phase approximation, are determined as ${W}_{\mu n,{\mu }^{{\prime} }m}=(\mu n|\mu {\prime} m)+{\sum }_{\alpha }{w}_{\mu n}^{\alpha }{w}_{{\mu }^{{\prime} }m}^{\alpha }[{(\omega -{\Omega }_{+}^{\alpha }+{\rm{i}}\eta )}^{-1}-{(\omega +{\Omega }_{+}^{\alpha }-{\rm{i}}\eta )}^{-1}]$ in the static approximation (*ω* = 0), where *w*^*Π*^ (equation (5)) is analogous to *w* but for electrons.

## Data Availability

Additional relevant data are available at 10.17034/04a9ffbe-e0c6-44e4-98bc-a2d30df3424c. Source data are provided with this paper.

## Code availability

The results presented in this study were generated using the EXCITON+program, which was developed by the Queen’s University Belfast group, adapting the EXCITON code (authored by C.H.P.) to incorporate positrons and extending its many-body theory capabilities. The version of EXCITON+ used to generate the current results is available at 10.17034/04a9ffbe-e0c6-44e4-98bc-a2d30df3424c. The latest EXCITON and EXCITON+ source codes are currently available under an Open Source license from C.H.P. (EXCITON) and D.G.G. (EXCITON+) on reasonable request, but we intend to detail them in subsequent publications.
